# A Comparison of Attitudes Toward Euthanasia Among Medical Students at Two Polish Universities

**DOI:** 10.1007/s13187-012-0414-4

**Published:** 2012-10-09

**Authors:** Wojciech Leppert, Leszek Gottwald, Mikolaj Majkowicz, Sylwia Kazmierczak-Lukaszewicz, Maria Forycka, Aleksandra Cialkowska-Rysz, Aleksandra Kotlinska-Lemieszek

**Affiliations:** 1 Chair and Department of Palliative Medicine, Poznan University of Medical Sciences, Osiedle Rusa 25 A, 61-245 Poznan, Poland; 2 Department of Radiotherapy, Nicolai Copernicus Hospital, Lodz, Poland; 3 Department of Quality of Life Research, Gdansk Medical University, Gdansk, Poland; 4 Department of Chemotherapy, Nicolai Copernicus Hospital, Lodz, Poland; 5 Department of Palliative Care, Chair of Oncology, Medical University, Lodz, Poland

**Keywords:** Ethics, Euthanasia, Medical students, Palliative medicine, Physician assisted suicide

## Abstract

The aim of the study conducted upon completion of obligatory palliative medicine courses among 588 medical students at two universities was to compare their attitudes toward euthanasia. Four hundred ninety-two (84.97 %) students were Catholics; 69 (11.73 %) declared they would practice euthanasia, 303 (51.53 %) would not, and 216 students (36.73 %) were not sure. The idea of euthanasia legalisation was supported by 174 (29.59 %) respondents, opposed by 277 (47.11 %), and 137 (23.30 %) were undecided. Five hundred fifty-six (94.56 %) students did not change their attitudes toward euthanasia after palliative medicine courses. Students from the two universities were found to have different opinions on practicing euthanasia, euthanasia law and possible abuse which might follow euthanasia legalisation, but they shared similar views on the choice of euthanasia if they themselves were incurably ill and the legalisation of euthanasia. Gender and religion influenced students’ answers. Differences observed between medical students at the two universities might be related to gender and cultural differences.

## Introduction

As a consequence of increased morbidity due to cancer and other chronic diseases as well as aging of the society, the number of patients requiring palliative care has increased. Medical students should be familiar with the basics of palliative medicine and end-of-life ethical principles [1]. Euthanasia and physician-assisted suicide (PAS) are widely discussed by medical professionals and in public debates with regard to their practice and legalisation [2]. Medical students’ attitudes toward euthanasia practice and law was explored in several studies.

Regarding euthanasia legalisation from 165 Swedish medical students of the first and the fifth years, 34 % expressed positive, 52 % negative opinion, and 13 % were undetermined; when discussing euthanasia for them in the future, 18 % ruled this out, 45 % considered they might do this, and 36 % were undetermined [3]. Among 160 fifth- and sixth-year Italian medical students, 50 % were against euthanasia legislation, 32 % for, and 18 % had no opinion; 50 and 57 % pointed out greater attention to quality of life and pain control, respectively, would eliminate the need for euthanasia and 60 % indicated religious beliefs and Hippocratic Oath may deter physicians from facilitating patient’s death [4]. In the US study of 166 medical students, respondents expressed opposition or uncertainty about death practices in five patient cases that illustrated severe suffering, especially opposition to their own involvement, and to non-physicians participation in assisted death [5]. Among 279 Puerto Rican medical students, 40 % supported euthanasia [6]. In a Polish study, 33.3 % of medical students supported the legalisation of euthanasia and over 20 % could not answer; 19.2 % students considered euthanasia in the case of their incurable illness, provided it was legal [7]. In our study among the third-year medical students who attended ethics lectures on the euthanasia practice and legalisation, respectively, 82 and 67 % of the surveyed responded negatively, 12 and 26 % positively, and 6 and 7 % did not answer [8].

There are no data on possible differences of medical students’ attitudes toward euthanasia between Polish universities and factors that may influence such potential differences. The aim of the study was to explore the knowledge of euthanasia and PAS definitions, attitudes toward euthanasia practice, law, and legalisation held by medical students from two Polish universities. Another aim was to check the impact of palliative medicine classes, place of residence, gender, and religion affiliation on students’ attitudes toward euthanasia and PAS.

## Methods

The questionnaire survey was conducted in the years 2008–2009 among medical students at two (Poznan and Lodz) medical universities. The written questionnaires were filled in voluntarily and anonymously upon the completion of obligatory palliative medicine courses that comprised 30 h in Poznan (for fifth- and sixth-year medical students) and Lodz (for sixth-year students). During theoretical lectures, seminars and practical classes students at both universities were provided with basic knowledge on symptom management as well as on psychological, social, and spiritual support. However, during palliative medicine courses, there was no specific discussion on euthanasia in either of the two medical universities.

The 12-question questionnaire (please see the Appendix) was based on our previous survey on breaking bad news, the euthanasia and PAS practice and legalisation conducted among third-year medical students and residents during internal medicine training [8], and a German study on euthanasia performed among medical students [9]. The pilot-testing of the questionnaire on 25 sixth-year medical students demonstrated that the questionnaire was well accepted and understood. The data were analysed with the licensed statistical package Statistica PL, version 8.0® and StatsDirect statistical software, version 2,6,5® (2007-11-12). The statistical evaluation of the demographic data, answers to questions and the differences between the surveyed from the two universities were based on a Chi-square test; with the *p* value of <0.05 being considered significant. The Local Bioethics Committees at Poznan and Lodz Medical Universities waived the need for the approval of the study protocol as the questionnaire survey did not concern patients.

## Results

From 650 students who were invited, 588 (90.5 %) participated in the study; 37 (9.5 %) out of 390 from Poznan and 25 (9.6 %) out of 260 from Lodz refused to participate. Among the 588 students who agreed to do so, there were 353 students (187 in the fifth and 166 in the sixth years) from Poznan and 235 students (in their sixth year) from Lodz. Students’ demographic data are shown in Table 1. More students from Lodz University lived in cities with over 500,000 inhabitants. A significant difference in the age between universities was also found. However, due to a very small standard deviation it did not matter for the interpretation of other variables. More women attended Lodz University. A similar structure of Catholics and atheists at both universities was found.

**Table 1:** Demographic characteristics of medical students

	Total	Medical University		*p* value
		Poznan	Lodz	
Number of students	588 (100 %)	353 (60.03 %)	235 (39.97 %)	
Place of residence				
Village	75 (12.76 %)	55 (15.58 %)	20 (8.51 %)	<0.001*
City^a^				
Up to 50	124 (21.09 %)	94 (26.63 %)	30 (12.77 %)	
50–100	71 (12.07 %)	35 (9.92 %)	36 (15.32 %)	
100–200	49 (8.33 %)	29 (8.22 %)	20 (8.51 %)	
200–500	40 (6.80 %)	26 (7.37 %)	14 (5.96 %)	
Over 500	229 (38.95 %)	114 (32.29 %)	115 (48.94 %)	
Age (mean ± SD)	24.44 ± 1.13	24.17 ± 1.17	24.84 ± 0.93	<0.001**
Gender				
Men	192 (32.65 %)	127 (35.98 %)	65 (27.66 %)	<0.05***
Women	396 (67.35 %)	226 (64.02 %)	170 (72.34 %)	
Religion				
Roman Catholic	492 (83.67 %)	289 (81.87 %)	203 (86.38 %)	0.324****
Atheist	87 (14.80 %)	56 (15.86 %)	31 (13.19 %)	
Other	9 (1.53 %)	8 (2.27 %)	1 (0.43 %)	

^a^Thousand inhabitants

*p < 0.001 (Chi-square test for a linear trend (M^2^) = 21.46; *df* = 1); **p < 0.001 (Student’s *t* test for non-paired data); ***p < 0.05 (Chi-square = 4.438; *df* = 1); ****p = 0.324 (Chi-square = 0.972; *df* = 1)

## Students’ Knowledge of Definitions and Opinions on Euthanasia Practice, Law, and Legalisation

**Fig. 1:**
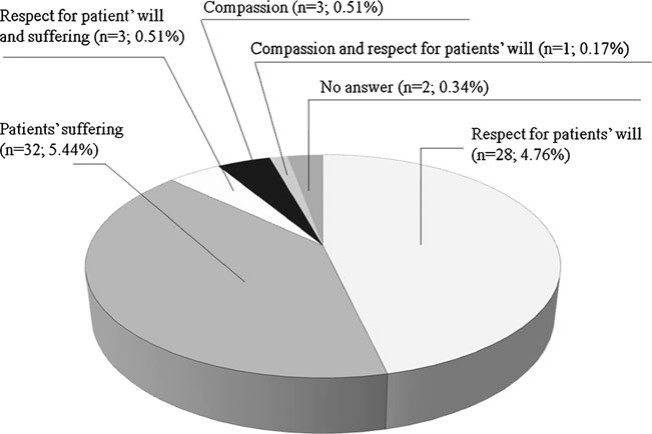
The arguments in favour of the euthanasia practice from 69 students (11.73 %)

**Fig. 2:**
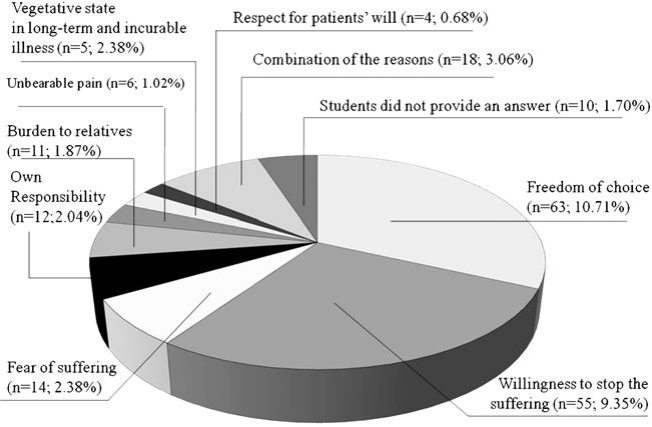
The arguments justifying 198 (33.85 %) students’ choice of euthanasia or PAS for themselves in case of their incurable disease

Both the euthanasia and PAS definitions were known to 468 (79.59 %) respondents, one definition to 84 (14.29 %), and 36 (6.12 %) respondents were not familiar with either of them. Students’ opinion on the euthanasia or PAS practice is shown in Table 2. The students’ arguments in favour of the euthanasia practice (*n* = 69) are listed on Fig. 1. The results of students’ choices when faced with an incurable disease of their own or one of their relatives are shown in Table 2. When the answer was euthanasia or PAS (*n* = 198), the arguments justifying it are shown on Fig. 2. The results of students’ answers regarding the euthanasia and PAS law and the legalisation of euthanasia or PAS are shown in Table 3. The euthanasia or PAS legalisation could lead to abuse according to 481 (81.80 %) students, 69 (11.73 %) of them responded it would not, and 38 (6.46 %) that they did not know.

**Table 2:** The declared practice of euthanasia and the choice in the case of incurable disease

Would you make a decision to perform euthanasia or assisted suicide?	Medical University		*p* value (Chi-square test)
	Poznan *n* = 353 (60.03 %)	Lodz *n* = 235 (39.97 %)	
Yes (69 (11.73 %))	56 (15.86 %)	13 (5.53 %)	<0.001
No (303 (51.54 %))	173 (49.01 %)	130 (55.32 %)	
Do not know (216 (36.73 %))	124 (35.13 %)	92 (39.15 %)	
If you or a close person of yours suffered from an incurable disease, would you like to have the right to choose?			
Natural death (355 (60.38 %))	205 (58.07 %)	150 (63.83 %)	0.08
Euthanasia (101 (17.35 %))	68 (19.26 %)	33 (14.04 %)	
Assisted suicide (97 (16.50 %))	54 (15.30 %)	43 (18.30 %)	
Do not know (35 (5.77 %))	26 (7.37 %)	9 (3.83 %)

**Table 3:** The declared support for current Polish law and the legalisation of euthanasia

Do you judge the law^a^ regulations forbidding euthanasia in Poland as^b^	Medical University		*p* value (Chi-square test)
	Poznan *n* = 353 (60.03 %)	Lodz *n* = 235 (39.97 %)	
Appropriate (330 (56.12 %))	176 (51.76 %)	154 (66.96 %)	<0.001
Insufficiently protecting from euthanasia and assisted suicide practice (83 (14.12 %))	49 (14.42 %)	34 (14.78 %)	
Too restrictive and should be ameliorated (157 (26.70 %))	115 (33.82 %)	42 (18.26 %)	
Are you in favour of legalisation of euthanasia or assisted suicide?			
Yes (174 (29.59 %))	116 (32.86 %)	58 (24.68 %)	0.1
No (277 (47.11 %))	158 (44.76 %)	119 (50.64 %)	
Do not know (137 (23.30 %))	79 (22.38 %)	58 (24.68 %)	

^a^The sentence in Poland for both euthanasia and assisted suicide is from 3 months to 5 years of imprisonment

^b^Please note that 18 (3.06 %; Lodz *n* = 5 and Poznan *n* = 13) students did not answer the question

## Differences Between Universities and Factors Influencing Students’ Answers

The differences between universities were significant with regard to students’ opinion on the euthanasia and PAS practice (Table 2), the euthanasia and PAS law (Table 3), and the suggestion that euthanasia or PAS legalisation could lead to abuse (*p* = 0.001). However, the differences between universities were not significant with respect to students’ choices when faced with an incurable disease of their own or one of their relatives (Table 2) and students’ answers on the legalisation of euthanasia or PAS (Table 3). Students’ answers regarding the practice and legalisation of euthanasia (Tables 4 and 5) and the euthanasia practice in the case of their own or their relatives’ incurable disease differed depending on the gender (*p* = 0.017) and religion (*p* < 0.001) but not on size of the place of residence.

**Table 4:** The declared practice and legalisation of euthanasia and gender

Would you make a decision to perform euthanasia or assisted suicide?	Gender		*p* value (Chi-square test)
	Men *n* = 192 (32.65 %)	Women *n* = 396 (67.35 %)	
Yes (69 (11.73 %))	41 (21.35 %)	28 (7.07 %)	<0.001
No (303 (51.53 %))	91 (47.40 %)	212 (53.54 %)	
Do not know (216 (36.73 %))	60 (31.25 %)	156 (39.39 %)	
Are you in favour of legalisation of euthanasia or assisted suicide?			
Yes (174 (29.59 %))	75 (39.06 %)	99 (25.00 %)	<0.001
No (277 (47.11 %))	84 (43.75 %)	193 (48.74 %)	
Do not know (137 (23.30 %))	33 (17.19 %)	104 (26.26 %)

**Table 5:** The declared practice and legalisation of euthanasia and religion

Would you make a decision to perform euthanasia or assisted suicide?	Religion^a^		*p* value (Chi-square test)
	Atheist *n* = 87 (15.03 %)	Catholic *n* = 492 (84.97 %)	
Yes (69 (11.73 %))	22 (25.29 %)	45 (9.15 %)	<0.001
No (303 (51.53 %))	22 (25.29 %)	274 (55.69 %)	
Do not know (216 (36.73 %))	43 (49.42 %)	173 (35.16 %)	
Are you in favour of legalisation of euthanasia or assisted suicide?			
Yes (174 (29.59 %))	45 (51.72 %)	125 (25.41 %)	<0.001
No (277 (47.11 %))	20 (22.99 %)	253 (51.42 %)	
Do not know (137 (23.30 %))	22 (25.29 %)	114 (23.17 %)	

^a^Please note that nine (1.53 %) students had other religious affiliations

## The Impact of Palliative Medicine Classes on Students’ Views and the Courses Evaluation

After palliative medicine classes, 23 (3.91 %) students changed their views on euthanasia and became its opponents, 9 (1.53 %) of them became its proponents, and 556 (94.56 %) did not change their views. However, the opinion that the number of patients’ requests for euthanasia could be limited by the introduction of palliative care was shared by 535 (90.99 %) respondents, the opposite view was expressed by 9 (1.53 %), 30 of the surveyed (5.1 %) responded that they did not know, and 14 (2.38 %) did not provide an answer.

**Fig. 3:**
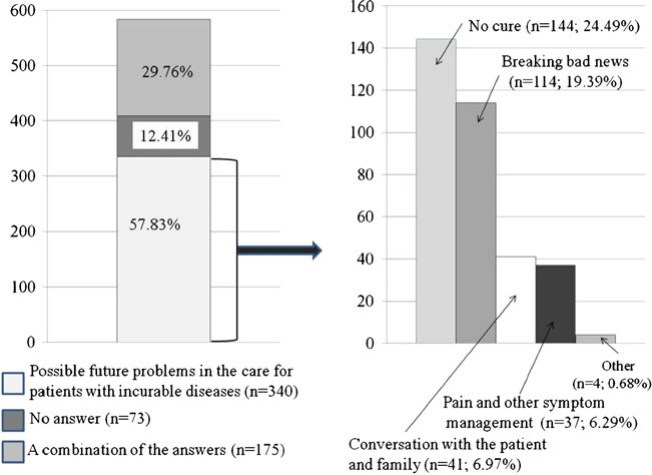
Possible students’ problems encountered in the future care for patients with incurable diseases

When asked if palliative medicine classes were helpful in the future care for patients, 250 (42.52 %) students responded yes to a significant extent, 229 (38.95 %)—to some extent, 88 (14.97 %)—to an unsatisfactory extent, 14 (2.38 %)—not at all, and 7 (1.19 %) students did not provide an answer. The answers to the question about the problems that the students might encounter in the future when dealing with patients with incurable diseases are shown on Fig. 3.

## Discussion

The study was conducted among 588 fifth- and sixth-year medical students at two Polish universities with above 90 % respond rate. A total of 80 % of respondents knew the definitions of euthanasia and PAS according to the EAPC [1]. The majority would not perform euthanasia or PAS (52 %), with 37 % being undecided. The most frequent reasons for the euthanasia practice were the respect for patients’ will and the need to stop patients’ suffering. Euthanasia practice opponents (52 %) corresponded with 59 % of students who would choose natural death in the case of their own or their relatives’ incurable disease. However, significantly more students would choose euthanasia or PAS (34 %) for themselves or relatives than perform it on patients (12 %). It is possible that the decision concerning patients is more difficult as the “do not know” response provided 37 % of the students regarding the euthanasia conducted on patients and 6 % referring to the euthanasia for them.

With respect to the euthanasia legalisation, 47 % of the opponents corresponded with 56 % of the students who supported the present law on euthanasia and PAS. Although the legalisation of euthanasia or PAS was supported by 30 % of the students and 23 % of them did not answer the question, over 80 % of the students thought that the euthanasia or PAS legalisation might lead to abuse. It may suggest that although the students who agree or are uncertain about the legalisation of euthanasia are nonetheless concerned about the possible abuse in this situation. This is in agreement with a German study in which 72–78 % of students thought that euthanasia legalisation could lead to abuse [9]. However, in a comparative study, the acceptance for euthanasia among the first-year Polish medical students was significantly lower (48 %) than Swedish (61 %; *p* < 0.007) and German (82 %; *p* < 0.0001) students [10].

Palliative medicine classes had a limited impact on students’ attitudes toward euthanasia as 4 % of them became euthanasia opponents and 95 % did not change their views. It may suggest that medical students’ views are rather stable and associated with their value systems. The percentage of euthanasia proponents among students decreases with years of medical training [11]. The impact of palliative medicine teaching was demonstrated in Germany as the percentage of euthanasia practice proponents, and those who would agree to be euthanised significantly decreased between the second- and sixth-year students at the university where palliative care was taught comparing to the university where there was no palliative care program [12]. In the Polish study among 431 medical students, when palliative care was presented as an alternative to euthanasia before the lecture, 39 % were for, 11 % in doubt, and 50 % against euthanasia; after the lecture, 29, 8, and 63 %, respectively [13]. However, during our courses euthanasia was not discussed.

During palliative medicine programs at both universities, students visit palliative in- and out-patient clinics and have direct contact with patients that may decrease the percentage of euthanasia proponents among physicians and medical students [14, 15]. A total of 91 % of our students indicated a possibility of limiting euthanasia requests by the introduction of palliative care into clinical practice; for comparison, only 30–45 % of German students expressed similar views [9]. Furthermore, 82 % of our respondents appreciated palliative medicine classes as helpful in the future care of patients with incurable diseases to a significant (43 %) or to some extent (39 %). Our students also expressed problems anticipated in their future work with incurable patients which mainly refer to the lack of the possibility to cure patients and psychological issues indicating a need for a better palliative medicine curricula development [16].

Students’ answers regarding the euthanasia practice and legalisation and their choice when facing incurable disease were varied with respect to gender and religion but not the size of the place of residence; the majority of respondents were women (67 %) and Catholics (85 %). The results are probably influenced by the Catholic Church and the statements of the late Polish Pope John Paul II [17], legal regulations, the Hippocratic Oath, and the Code of Medical Ethics. The impact of faith was demonstrated in the case of Norwegian medical students as the percentage of Catholics supporting the euthanasia and PAS practice was lower in comparison with atheists and other faiths [18]. Religiosity is a predictor of a negative attitude towards euthanasia among UK doctors [19]. The gender impact (males supported legalising euthanasia more often than females) was found among Finnish physicians [20].

The differences between universities were found as regards the euthanasia practice, law, and possible abuses if it was legal, but not choices in the case of own incurable disease and the legalisation of euthanasia. The fact that more students from Poznan University supported the euthanasia practice and revision in the law and were less afraid of abuse in case euthanasia is legal may be attributed to a higher percentage of women at Lodz University and hypothetically to cultural differences as central Poland (Lodz) is more often associated with traditional values than the more liberal western part (Poznan) which seems to be rather similar to Western Europe [21]. These differences need further elucidation which also applies to similar results obtained on euthanasia legalisation and practice on students themselves.

The limitations of the study comprise only a single questionnaire administration upon courses completion. Several questions referred to hypothetical situations such as euthanasia practice on patients or on students themselves. The number of responses to each question was compared with only partially explored students’ motives. No other factors that potentially might influence students’ responses such as personality, value system, and emotional state were assessed. Combining the responses from the fifth- and sixth-year students in Poznan and different curricula in Poznan and in Lodz might have influenced the results.

In conclusion, the majority of Polish medical students do not support euthanasia practice, change in law that bans euthanasia, and to less extent its legalisation with significant percentage being undecided; most students expressed concerns of abuse if euthanasia was legal. Palliative medicine classes had little influence on students’ views. Differences exist between the two universities in students’ attitudes toward euthanasia and PAS practice, law, and possible abuse in case it is legal; gender, religion, and probably culture but not size of the place of residence influenced the results. Future studies may explore more precisely factors that influence Polish medical students’ attitudes toward euthanasia and PAS.

## Appendix

### Questionnaire

We kindly ask you to answer honestly the questions below by circling the appropriate answer. Under each answer you may also give your explanation. This questionnaire is voluntary and anonymous.

Date:

Faculty:

Year of studies:

Gender: woman/man

Age:

Religion:

Place of residence (permanent):

1. Village
2. Town to 50,000
3. Town of 50–100,000
4. City of 100–200,000
5. City of 200–500,000
6. City of over 500,000 inhabitants

Definitions according to the EAPC Ethics Task Force, *Palliative Medicine* 2003; 17 (2): 97–101:

Euthanasia is killing on request and is defined as a doctor intentionally killing a person by the administration of drugs at that person’s voluntary and competent request.

Physician-assisted suicide is defined as a doctor intentionally helping a person to commit suicide by providing drugs for self-administration, at that person’s voluntary and competent request.

1. Do you know these definitions:
  A. Yes, both
  B. Yes, one (please write which one):
  C. No.
2. Would you make a decision of committing an act of euthanasia or assisted suicide?
  A. Yes
  B. No
  C. I do not know.
3. If the answer in question 2 is yes, what would be the reason(s) of your decision:
4. If you or a close person of yours suffered from an incurable disease, would you like to have the right to choose:
  A. Natural death
  B. Euthanasia
  C. Assisted suicide.
5. If your choice in question 4 is B or C what would be the reason(s) of your decision:
6. According to the 150th article, 1§ of the Polish Criminal Code: A person performing mercy killing of another person on his/her request is subject to the imprisonment from 3 months to 5 years; § 2: In exceptional cases the court may alleviate or even completely refrain from the punishment. Do you judge these law regulations as:
  A. Appropriate
  B. Insufficiently protecting from the euthanasia and assisted suicide practice
  C. Too restrictive and they should be ameliorated.
7. Are you in favour of the legalisation of euthanasia or assisted suicide?
  A. Yes
  B. No
  C. I do not know.
8. Do you think that legalisation of euthanasia or assisted suicide could lead to abuse:
  A. Yes
  B. No
  C. I do not know.
9. In what way have palliative medicine classes influenced your attitude towards euthanasia:
  A. Previously I was a euthanasia proponent and now I am a euthanasia opponent
  B. Previously I was a euthanasia opponent and now I am a euthanasia proponent
  C. Did not change my attitude with this regard.
10. Do you think that by introducing palliative care (appropriate treatment of pain and other symptoms, psychological, social and spiritual support) it is possible to effectively prevent or limit euthanasia requests from patients with incurable diseases:
  A. Yes
  B. No
  C. I do not know.
11. Were palliative care classes helpful in preparing you to care for patients with incurable diseases:
  A. To a significant extent
  B. To some extent
  C. To an unsatisfactory extent
  D. Not at all.
12. Which of the problems discussed during palliative care classes may be difficult for you in the future contact with patients diagnosed with incurable diseases:
  A. Pain and other symptoms treatment
  B. Breaking bad news to patients regarding diagnosis and prognosis
  C. Lack of the possibility to cure the patient
  D. A conversation with a patient’s family
  E. Other (please list)

Notes and commentaries:

Thank you very much for your help!
